# Fly navigational responses exploit plume-specific odor motion and gradient cues

**DOI:** 10.1073/pnas.2608896123

**Published:** 2026-07-14

**Authors:** Samuel Brudner, Baohua Zhou, Viraaj Jayaram, Gustavo Madeira Santana, John P. Crimaldi, Damon A. Clark, Thierry Emonet

**Affiliations:** ^a^ Department of Molecular Cellular and Developmental Biology, Yale University, New Haven, CT 06511; ^b^ Quantitative Biology Institute, Yale University, New Haven, CT 06511; ^c^ Department of Physics, Yale University, New Haven, CT 06511; ^d^ Department of Neuroscience, Yale University, New Haven, CT 06510; ^e^ Wu Tsai Institute, Yale University, New Haven, CT 06510; ^f^ Department of Civil, Environmental and Architectural Engineering, University of Colorado, Boulder, CO 80309

**Keywords:** navigation, olfaction, behavioral optimization, *Drosophila*, sensory processing

## Abstract

Odor cues guide animals to food and mates. Different environmental conditions can create differently patterned odor plumes, making navigation more challenging. Prior work has shown that animals turn upwind when they detect odor and cast crosswind when they lose it. Animals with bilateral olfactory sensors can also detect directional odor cues, such as odor gradient and odor motion. It remains unknown how animals use these two directional odor cues to guide crosswind navigation in odor plumes with distinct statistics. We investigated this problem theoretically and experimentally. Here, we show that these directional odor cues provide complementary information for navigation in different plume environments. We numerically analyzed experimentally generated plumes to show that odor gradient cues are more informative about crosswind directions in a smooth odor plume, while odor motion cues are more informative in a complex plume. Neural networks trained to optimize crosswind turning converged to distinctive network structures that are tuned to odor gradient cues in a smooth plume and to odor motion cues in a complex one. These trained networks improved the performance of artificial agents navigating plume environments that match the training environment. Last, we recorded *Drosophila* fruit flies as they navigated these odor plume environments. In the smooth plume, the gradient cue was the significant predictor of fly crosswind turning; in the complex plume, the motion cue was the significant predictor. Overall, these results demonstrate that these directional odor cues are complementary across environments and that animals exploit this relationship to navigate plumes with different statistics.

## Significance

Many animals navigate diverse odor plumes by smell. While upwind movement upon odor detection is well established, less is known about how animals steer crosswind to stay in the plume. We show that directional odor cues—gradient and motion—guide crosswind navigation differently in two plumes with different structures: one in which odor concentration smoothly decreased away from the centerline and another in which odor filaments moved outward from the centerline. Neural networks trained to optimize crosswind navigation developed stronger gradient sensitivity in the former and stronger motion sensitivity in the latter. Experimentally, the contextually informative cue best predicted the turning decisions of fruit flies. These findings may generalize to other animals equipped with bilateral sensors navigating odor plumes.

**Fig. 1.**
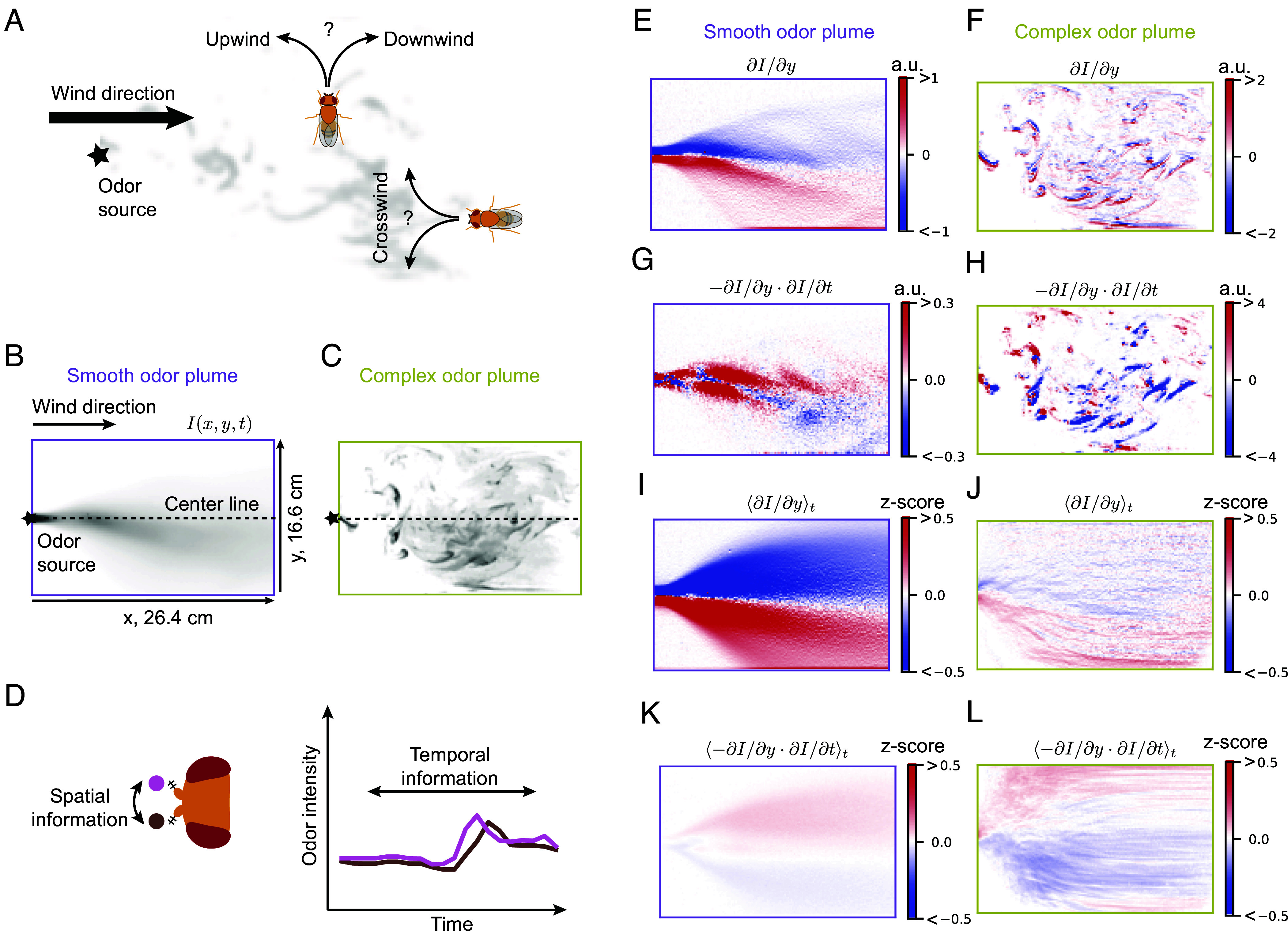
Directional information for navigation in different plume environments. (*A*) A fly needs to use various directional features to find the odor source (e.g., food). In a 2d environment, flies need to navigate both in the upwind/downwind and in the crosswind directions. Wind direction breaks symmetry in the upwind/downwind axis but does not distinguish crosswind directions. (*B* and *C*) Snapshots of two typical plume environments that a fly may encounter: a smooth odor plume and a complex odor plume. The odor intensity is represented by I(x,y,t). The upwind/downwind and crosswind axes are labeled x and y. (*D*) the fly’s two antennae can detect spatiotemporal information of the odor plume. (*E* and *F*) Gradient value in the y dimension in the two plume types. (*G* and *H*) Motion value in the y dimension in the two plume types (*SI Appendix*, *Supplementary Methods*). (*I* and *J*) Time-averaged, z-scored gradient value in the y dimension. (*K* and *L*) Time-averaged, z-scored motion information in the y dimension (*SI Appendix*, *Supplementary Methods*).

The sense of smell is critical for animals to locate food and find mates (1–16). To succeed in these tasks, they detect odor and wind patterns that inform their navigation choices (Fig. 1*A*) (17–19). Multiple physical processes generate these sensory patterns. Odor molecules disperse from a source via molecular diffusion and are carried by the wind as odor plumes. Fluctuations in the speed and direction of the wind and other environmental factors such as surface roughness, obstacles (e.g., grass, trees, pebbles), and odor source motion affect the spatiotemporal structure of these plumes and, consequently, the sensory patterns available to navigating animals (2, 20–23). In vision, there is evidence that statistical properties of visual scenes strongly influence how visual neural circuits process input signals (24, 25). Different plume statistics could also convey different types of information for olfactory navigation, and navigation behavior may reflect these differences. Here, we address this question using fruit flies to examine how they use distinct bilateral olfactory cues when navigating odor plumes with different spatiotemporal structure.

Measuring odor concentration as a function of both space and time is extremely difficult. Consequently, most field measurements of odor plumes only characterize the temporal statistics of the signal in one location at a time. While these recordings demonstrate large variation in the temporal statistics of odor signals across environments (2, 20–22, 26), the spatiotemporal statistics required to assess bilateral directional cues like gradient and motion remain largely unknown. Laboratory experiments (27–30), theory (31, 32), and simulations (27, 33–35) provide guidance and illustrate how, across conditions, various mechanisms contribute differentially to the dispersion and broadening of an odor plume as it travels downwind, thereby generating plumes with different spatiotemporal statistics (30, 32). In weak, steady air flows and near surfaces, odor plumes spread by slow molecular diffusion and weak velocity fluctuations, resulting in odor concentrations that gradually decrease downwind from the source and outward away from the plume’s centerline [Fig. 1*B* and Movie S1 (30, 36)]. In nearly laminar wind, fluctuations from random shifts in wind direction, eddies, and boundary-layer detachment due to obstacles introduce bends, folds, flapping, and plume meandering (22, 31). Even before full turbulence, these perturbations stretch, fold, and intermittently fragment what was a continuous stream of odor into filaments interspersed with regions where signal falls below detection (2, 21). These filaments disperse in the crosswind direction (Fig. 1*C* and Movie S2). As wind forcing and fluctuations grow toward the turbulent regime, these stretching–breaking events amplify, and the plume becomes a sparse, intermittent assembly of concentrated filaments that evolve rapidly in time (3, 4, 8, 22, 24, 25, 31, 37, 38).

A basic strategy for navigating attractive odor plumes is to surge upwind when the odor is detected but to initiate a search when the signal is lost. Although search can have directed upwind or downwind components, its crosswind component has often been characterized as random casting that may allow the animal to reconnect with the plume, although recent work suggests these crosswind trajectories can possess more structure (3, 4, 20, 21, 28, 35, 36, 38–48). This surge-and-cast strategy has been extensively studied in theoretical models (35, 40, 43–45). A key feature of the strategy is that wind direction serves as the primary directional cue, while the odor signal itself only regulates when to surge upwind or cast crosswind. However, this approach has a significant limitation: Although the wind direction serves as a key directional cue, it does not help an animal facing upwind distinguish between turns toward or away from the odor source (Fig. 1*A*). In other words, the wind breaks directional symmetry in the upwind–downwind axis but not in the crosswind direction, which is critical for efficiently locating the odor source.

Animals might improve on the surge-and-cast strategy by extracting directional information from the odor signal, which could complement information provided by the wind direction. In fact, many animals orient relative to directional olfactory cues that they calculate by comparing signals across separated bilateral sensors, like antennae in insects and nostrils in vertebrates. The spatial separation between sensors makes it possible to measure odor concentration at two locations and compare them. Stronger attractive stimulation of the left (right) side can drive left (right) turning in invertebrates (49–52) and vertebrates (14, 53), a response to the instantaneous spatial concentration difference, or *odor gradient* (Fig. 1*D*). In contrast, temporal comparisons between the right and left signals can provide information about the motion of the odor across the sensors, or *odor motion*. Experiments have shown that sequential activation of olfactory receptors in the left-to-right or right-to-left direction drives left (right) turning in invertebrates (7, 27) and vertebrates (9, 54), constituting responses to odor motion across the sensors (Fig. 1*D*).

These two directional odor cues are well positioned to help navigators break symmetry perpendicular to the wind to distinguish between crosswind directions. In this way, they may help animals navigate toward plume centerlines (Fig. 1*A*) (7, 27). Since odors can disperse from the centerline differently across environments, we wondered how plume structure would influence strategies to navigate crosswind using directional odor cues. Here, we consider the roles of both odor gradient and odor motion signals in centerline-oriented navigation across two plumes chosen for their different spatiotemporal statistics. We hypothesized that odor gradients and odor motion would contribute differently to fly navigation in these two plumes and that fly navigation behaviors would be matched to these differences.

To quantify the reliability of odor gradients and odor motions as predictors of the direction to the plume centerline, we optimized shallow and interpretable neural network models to predict the centerline direction in each environment. These models showed differential sensitivity to odor gradient and motion cues that depended on their training environments. Using simulated agents, we demonstrated that using plume-appropriate directional odor cues improved navigation performance compared to using plume-inappropriate cues and compared to a strategy using wind as the only directional cue. Finally, we tracked *Drosophila* fruit flies navigating these two plumes and measured their crosswind navigation decisions. The fly behavioral responses to odor gradients and odor motion depended on the plume statistics and was consistent with potential strategies that account for the differences in cue reliability across the two plumes. Overall, we show that optimized behavioral strategies exploit different directional odor cues in two different environments and predict crosswind turning decisions by animals engaged in olfactory navigation.

## Results

### Directional Odor Features Have Plume-Specific, Antisymmetric Organization Around the Centerline.

The spatiotemporal features of the odor signals that flies encounter in nature are not precisely known. To investigate gradient and motion cues in navigation we used two recordings of real odor plumes that were generated using established experimental protocols and that have been used in the laboratory with fruit flies in vivo (27, 28, 30, 36). One plume, which we call *smooth*, was generated by releasing odor near a flat surface into homogenous turbulence. Another plume, which we call *complex,* was generated by releasing odor into a laminar wind tunnel used for walking fly experiments while flow was perturbed by stochastic lateral air jets (Fig. 1 *B* and *C*; see Movies S1 and S2 and *SI Appendix*, *Supplementary Methods*) (27, 30, 36). We stress that the two terms—smooth and complex—do not represent the two ends of a spectrum of plume structures that wild flies encounter, since those in natural spatiotemporal structures remain unmeasured. However, these two plumes are useful for studying how directional odor features—odor gradient and odor motion—can assist centerline-finding. They are wide, so that they accentuate the importance of centerline-finding for successful navigation. They also have distinct spatiotemporal structure. They are examples, respectively, of a continuous and of a more filamentous plume. As a result, they differ in temporal characteristics that have been studied in navigation (17, 18, 28, 36, 40, 43): The smooth plume contains more continuous odor signal, while the complex plume is intermittent with rapid fluctuations in intensity (*SI Appendix*, Fig. S1). We additionally analyzed odors released far from a boundary layer (freestream) into laminar wind at a parametrically varied range of wind speeds; the temporal statistics—encounter frequency and intermittency—span a range from smooth-like at low wind speeds to complex-like at high wind speeds. The cue antisymmetry index (*SI Appendix*, Fig. S1, column v; the log ratio of motion to gradient antisymmetric organization) increases across this range: Gradient-cue antisymmetric organization remains roughly invariant, while motion-cue strength and antisymmetric organization grow with wind speed (*SI Appendix*, Fig. S1; plumes generated in ref. 30). The spatiotemporal statistics of plumes in fully turbulent atmospheric or cluttered natural environments remain unmeasured at the resolution required to assess directional cue availability.

To assess differences in odor gradient and odor motion distributions between plumes, we quantified the strength of the odor gradient and odor motion in the (cross-wind) y direction by calculating ∂I/∂y and -∂I/∂y·∂I/∂t, respectively at each point (x,y,t) in each plume (snapshots in Fig. 1 *E*–*H*; see *SI Appendix*, *Supplementary Methods*). In the remainder of the paper, we will refer to these as the gradient and motion features. These features were organized in qualitatively different ways in the smooth and complex plumes (Fig. 1 *E*–*H*). In the smooth plume, the gradient feature had opposite signs on opposite sides of the centerline while in the complex plume, it did not exhibit this antisymmetric organization across the centerline. In contrast, the motion feature showed a different pattern of organization. In the smooth plume, there was no clear organization of the motion feature around the centerline, while in the complex plume the motion feature had opposite signs across the centerline. In summary, the gradient and motion features exhibited complementary distributions, with each organized antisymmetrically around the centerline in one plume type but not the other.

Plume structure varies over time, so snapshots are not fully representative. Therefore, we calculated the temporal average of each feature at each location and divided these averages by the feature SD to obtain z-scored values for each feature at each location (Fig. 1 *I*–*L*). These z-scores can be interpreted as the signal-to-noise of the mean of each feature. The averages of both features exhibit antisymmetric organization around the plume centerline. However, the odor gradients have higher z-scores in the smooth plume than in the complex one, while the odor motion has higher z-scores in the complex plume than in the smooth one. These results indicate that, in the smooth plume, antisymmetric organization of the odor gradient is more reliable, while in the complex plume, antisymmetric organization of the odor motion is more reliable. When we investigated gradient and motion features in freestream plumes at different wind speeds, we found that the strength and antisymmetric organization of the motion cue grew with increasing wind speeds, generating temporal statistics that increasingly resembled those in the complex plume (*SI Appendix*, Fig. S1 *A*–*E*). This result also confirms that the weak motion signal in the smooth plume is not due to lower sampling resolution of the smooth plume compared to the complex one: All freestream plumes and the smooth plume were sampled equally. Relatedly, the complex plume’s cue organization was robust to reduced sampling resolution, only changing when sampled more coarsely than the smooth plume (*SI Appendix*, Fig. S1 *F* and *G*).

### Plume-Specific Features Predict Centerline Direction.

**Fig. 2.**
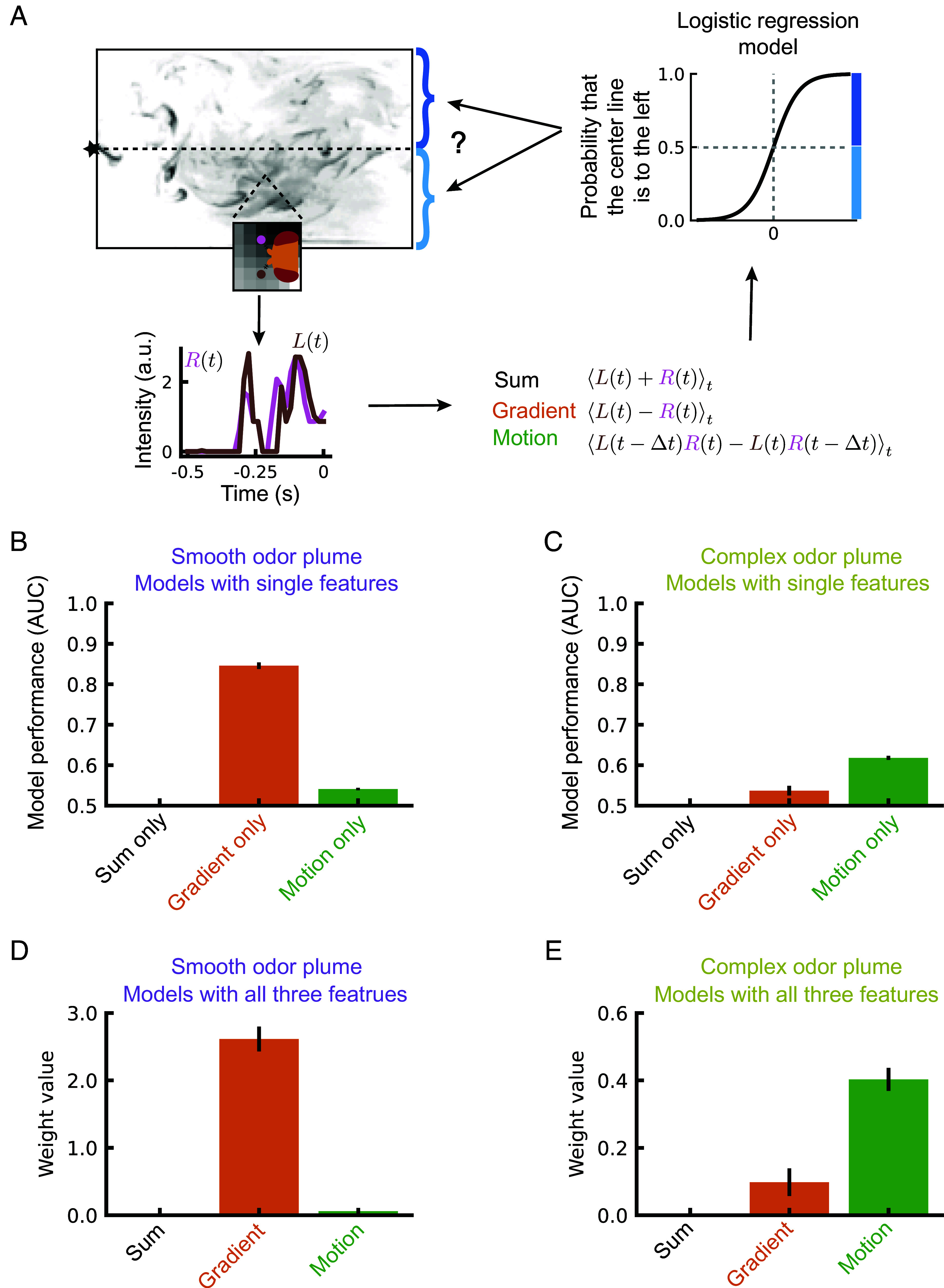
Gradient and motion information have different predictive powers in different plume types. (*A*) Three types of features are calculated from the two odor signals detected by the two antennae, R(t) and L(t): sum, gradient, and motion (*SI Appendix*, *Supplementary Methods*). 500 ms averages of feature traces are used to predict the direction to the centerline via logistic regression. (*B* and *C*) The three features are used individually to fit logistic regression models. ROC AUC are reported for the test samples. (*D* and *E*) The three features were used simultaneously to fit logistic regression models, and the trained weights are reported.

If a feature is organized antisymmetrically across the plume centerline, it can provide information about the crosswind direction toward the centerline. We sought to quantify the predictive relationships between these features and the centerline direction for each plume. In each plume, we trained logistic regression models to predict the centerline direction using these odor features and subsequently scored their performance. The data were sampled randomly throughout each plume, assuming that the bilateral sensors faced upwind. They sampled two temporal odor signals, R(t) and L(t), at two nearby locations, 0.46 mm apart, for a duration of 0.5 s (Fig. 2*A*). We engineered three different features: the odor sum, the odor gradient, and the odor motion, defined as $\langle L\left(t\right)+R\left(t\right)\rangle_{t}$, $\langle L\left(t\right)-R\left(t\right)\rangle_{t}$, and $\langle L\left(t-\Delta t\right)R\left(t\right)-L\left(t\right)R\left(t-\Delta t\right)\rangle_{t}$, respectively. The odor motion feature is the net spatiotemporal correlation in the odor intensity, with a delay of Δt≈0.017 s, or one time step in our recordings (*SI Appendix*, *Supplementary Methods*). This delay time is near the limit at which temporal jitter in the receptor neuron responses is estimated to degrade motion detection performance (~10 ms in ref. 27; longer delays are tested in *SI Appendix*, Fig. S2). Positive and negative net correlations indicate left-to-right and right–left odor motion. We trained our models to use these three features to best predict the probability that they are to the right or left of the centerline (Fig. 2*A*, see *SI Appendix*, *Supplementary Methods*).

To assay the predictive power of each feature in each plume type, first we trained a separate model for each odor feature in each plume type. We assessed the performance of each of these models using receiver operating curves (ROCs), which reflect the tradeoff between detections and false positives for binary classifiers. ROCs can be summarized by the ROC area-under-the-curve (AUC) value, a statistic that is 0.5 for random classifier performance and approaches 1 for perfect classification. Models that used odor gradient to predict the smooth plume centerline direction had higher AUCs than models that used odor motion for the same task (Fig. 2*B* and *SI Appendix*, Fig. S2*A*). This indicates that in the smooth plume, odor gradients were more predictive than odor motions of the centerline direction. In the complex plume, the opposite was true, so that odor motion was more predictive than odor gradients in determining the centerline direction (Fig. 2*C* and *SI Appendix*, Fig. S2*B*). In both plume conditions, the bilateral sum ROC AUC values were 0.5, indicating that this control feature, which contains no spatiotemporal information, had no predictive power (Fig. 2 *B* and *C*). In both plume types, odor motion features with one-frame delays had higher ROC AUC values than motion features using longer delays (*SI Appendix*, Fig. S2*C*). Together, these quantifications support our previous qualitative description of the spatial distributions of odor gradient and odor motion features in the two plumes (Fig. 1). Specifically, in the smooth plume odor gradients but not motions reliably indicate the direction to the centerline. Conversely, in the complex plume odor motions but not gradients reliably indicate the centerline direction.

These single-feature models are conceptually simple, but they do not capture the possibility that correlations between gradient and motion features enable *both* to contribute to centerline predictions when these features are weighted and combined. To assess this possibility, we trained logistic models to use linear combinations of the z-scored values of all three features. In this analysis, a model’s fitted weight for a feature quantifies the influence of variation in that feature on classification. For example, a feature weight of zero magnitude indicates that the model ignores the effect of this feature. The model trained in the smooth plume learned a larger weight for the odor gradient feature than for the odor motion feature (Fig. 2*D* and *SI Appendix*, Fig. S2*D*). Conversely, the model trained in the complex plume learned a larger weight for the odor motion feature than for the odor gradient feature (Fig. 2*E* and *SI Appendix*, Fig. S2*D*). In both plumes, the weights for the odor sum feature were 0, as expected. These results indicate that, in different plume environments, odor gradient and motion features have different predictive powers about the centerline direction, even when considering correlations between the features.

### Optimized Bilateral Computation for Centerline-Finding Recovers Plume-Specific Odor Features.

Our analysis so far considered features that we engineered by computing averages and correlations between simulated antennal inputs. A real fly, however, does not have direct access to such features. To exploit the asymmetry of the gradient and motion features during olfactory navigation, a fly must process right and left antennal signals to extract relevant information. To investigate what such processing would look like, we built neural network models, which we trained to use bilateral time series to perform the same binary classification of the centerline direction as our logistic models above. After training, we assessed how solutions differed between the two plumes, focusing on the trained networks’ sensitivity to odor gradient and motion features.

**Fig. 3.**
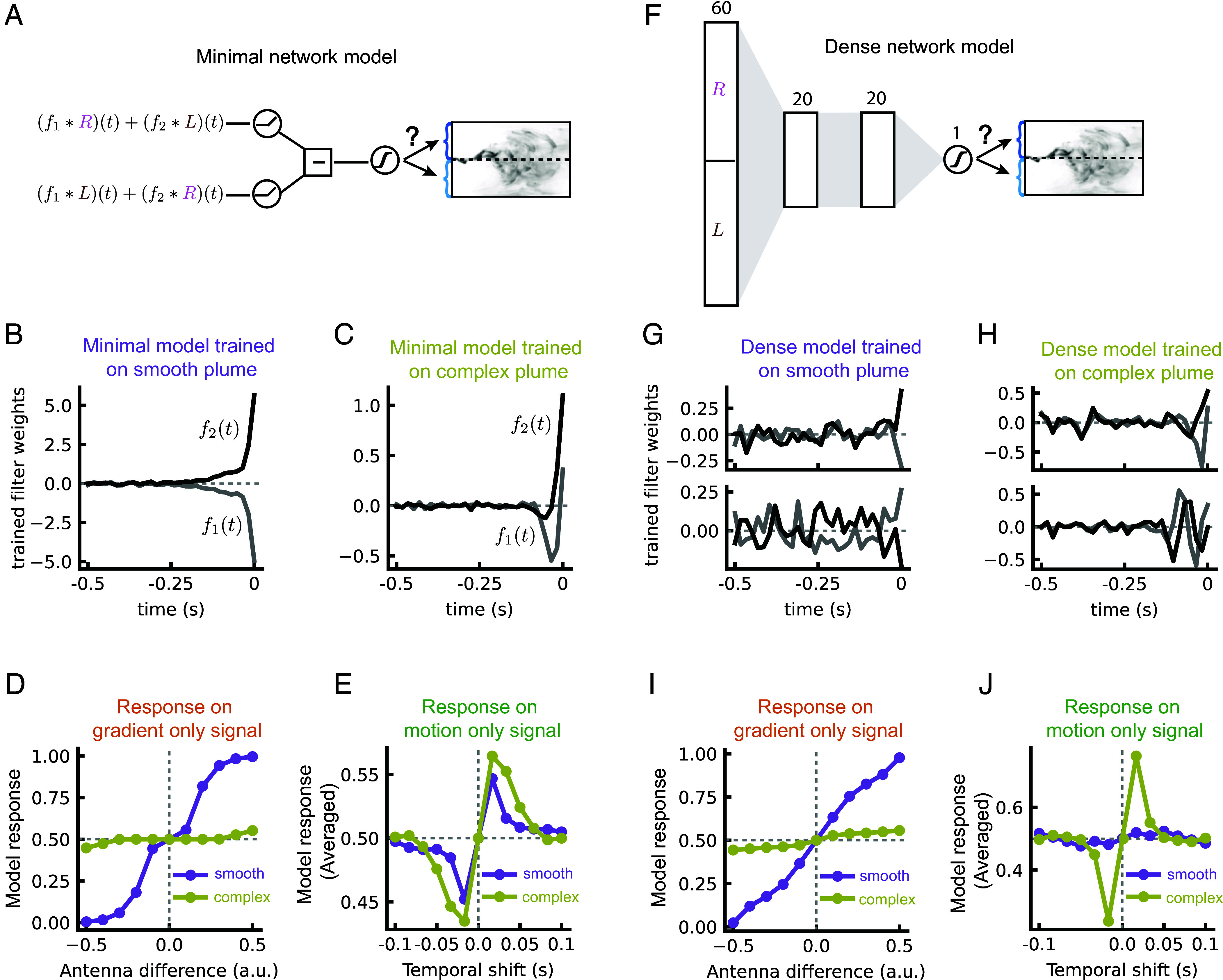
Neural network models trained in different plume types learn to sense different stimulus features. (*A*) A MNM only has two trainable filters, represented by $f_{1}\left(t\right)$ and $f_{2}\left(t\right)$, respectively. The two inputs, R(t) and L(t), are generated when the fly head undergoes a rotational random walk (*SI Appendix*, Fig. S3 *A* and *B*). The output unit is a sigmoid function, which yields a probability that the centerline is to one side of the fly. (*B*) Trained filters of the MNM in the smooth odor plume, represented by the gray and black lines, respectively. (*C*) Trained filters of the MNM in the complex odor plume, represented by the gray and black lines, respectively. (*D*) Responses of the trained MNMs to gradient stimuli as a function of the antennal difference (*SI Appendix*, *Supplementary Methods*). (*E*) Responses of the trained MNMs to motion stimuli as a function of the temporal shift between the two input signals (*SI Appendix*, *Supplementary Methods*). (*F*) A DNM has two hidden layers, each with 20 units. The feedforward connections between the input layers (by concatenating the two inputs), the first hidden layer, and the second hidden layer are all-to-all. The output unit is a sigmoid function, which gives a probability that the centerline is to one side of the fly. (*G*) Examples of first layer filters trained in the smooth plume between the input layer and the first hidden layer (2 out of 20). The black lines correspond to the filters acting on L(t) while the gray lines correspond to the filters acting on R(t). (*H*) As in (*G*), but for filters trained in the complex plume. (*I*) As in (*D*), but for the DNMs. (*J*) As in (*E*), but for the DNMs.

First, we built a minimal network model (MNM) that has only two sets of trainable parameters, $f_{1}\left(t\right)$ and $f_{2}\left(t\right)$, the temporal filters acting on the odor signals at two simulated antennae (including an intercept parameter) (Fig. 3*A*). The MNM convolves these two temporal filters with the two odor signals, R(t) and L(t), sums these products, and passes the sum through a nonlinear activation function (ReLU). To incorporate left–right antisymmetry in the model, we introduced an opponent structure to the model by subtracting a mirror-symmetric version of the same computation (Fig. 3*A*). Additionally, since real navigators make turns that alter the orientation of their sensors relative to their environment, the orientation of the artificial fly underwent a Wiener process but faced upwind at time 0, the most recent time in each training sample (*SI Appendix*, Fig. S3 *A* and *B* and *Supplementary Methods*), a technique that ensures that information in the distant past is not relevant to the inference (55, 56).

We trained two MNMs to identify the centerline direction in each plume type (*SI Appendix*, Fig. S3 *C*–*E*). The resulting filters had very different structures in the two plumes. For models trained in the smooth plume, the filters acted like a gradient detector (Fig. 3*B*), computing the difference in intensity between the two antennae. For models trained in the complex plume, filters were more complex (Fig. 3*C* and *SI Appendix*, Fig. S3*D*). The opposite signs of the filters suggest that they are also sensitive to gradients, but the delayed, negative lobe in $f_{1}\left(t\right)$ is reminiscent of a visual motion detection model proposed based on data in the rabbit retina (57), for which the delayed inhibition in $f_{1}\left(t\right)$ tends to veto signals when they arrive in the nonpreferred order.

To better quantify the response properties of each model, we created synthetic signals containing exclusively gradient or motion content (*SI Appendix*, Fig. S3 *F* and *G*). For the gradient content, we varied the difference in intensity between the two antennal inputs (*SI Appendix*, *Supplementary Methods*). For the motion content, we generated binary signals and enforced a correlation between the two antenna signals with different delays (*SI Appendix*, *Supplementary Methods*) (58). The model trained in the smooth plume responded more strongly to the gradient signals than the model trained in the complex plume (Fig. 3*D*). Interestingly, the models trained in the complex and smooth plumes both responded strongly to the motion signals with various delays (Fig. 3*E*). The gradient detector obtained from training in the smooth plume responds to the motion signals because of small temporal asymmetries between the two filters (Fig. 3*B*). Responses to the motion signals disappear if exactly symmetrical filters are used (*SI Appendix*, Fig. S3*H*).

The minimal models are interpretable, but not very expressive. More expressive models could allow us to assay the behavior of algorithms—particularly their responses to gradients and motion—closer to the upper limit of task performance. We therefore built a general dense network model (DNM) with the same opponency structure (*SI Appendix*, *Supplementary Methods*) to predict the centerline direction (Fig. 3*F* and *SI Appendix*, Fig. S3 *I*–*K*). This dense model employs 20 sets of temporal filters between the input layer and the first hidden layer (Fig. 3 *G* and *H*), but only performs modestly better than the MNM (*SI Appendix*, Fig. S3*K*). Interestingly, the results with these more expressive models are similar to the results with the MNM. The temporal filters trained in the smooth plume look more like gradient detectors, while those trained in the complex plume look more like motion detectors, although in some cases the distinction was less clear (*Bottom* row in Fig. 3 *G* and *H*). To evaluate the sensitivity of these models to odor gradients and odor motion, we tested the trained DNMs on the same synthetic data as before (*SI Appendix*, Fig. S3 *F* and *G*). When tested on the gradient signals, the model trained in the smooth plume was more sensitive to gradient signals compared to the model trained in the complex plume (Fig. 3*I*), similar to the results with MNMs. On the other hand, while the MNMs trained in the complex and smooth plumes responded to the motion signals with similar strength, the DNM trained in the complex plumes responded more strongly to the motion signals than the one trained in the smooth plumes (Fig. 3*J*). These results suggest that increased flexibility afforded by the dense architecture allowed the models to become even more specialized for gradient and motion features when trained in the smooth and complex plume respectively.

### Sensitivity to Plume-Appropriate Directional Features Enhances Agent Navigation.

Our previous results indicate that different plume environments invite different computations to predict the centerline direction. To support navigation, responses to these features must be integrated with broader strategies to reach the odor source. Critically, they must be combined with odor timing cues that regulate turning in the upwind or downwind direction and tend to be symmetrically organized around the plume centerline (28, 34, 36, 59, 60). We hypothesized that improved performance from bilateral sensing would depend on the same correspondence between specific directional odor features and plume types identified in our earlier analysis of centerline prediction.

To test this hypothesis, we built artificial agents that navigated both plumes using different directional odor features and we quantified their ability to locate the odor source. As a null navigational model for evaluating the contributions of odor gradient and motion sensing, we repurposed a previously published navigational model that relies solely on wind direction and the timing of odor encounters to bias turns upwind. Experiments have shown that walking flies navigating real plumes with statistics similar to the smooth and complex plumes bias their upwind turns according to the duration and frequency of odor encounters (28, 36). Subsequent theoretical work showed that both classes of temporal cues are required for robust navigation in both plumes (43) and that an agent-based model that responds to both encounter duration and frequency can recapitulate fly behavior across a wide range of optogenetically controlled odor durations and frequencies (40). This model, hereafter called the *wind-only* model, performs similarly in the smooth and complex plumes (see below). It therefore provides an appropriate baseline for assessing the additional contributions of directional odor cues across plumes with different temporal statistics.

**Fig. 4.**
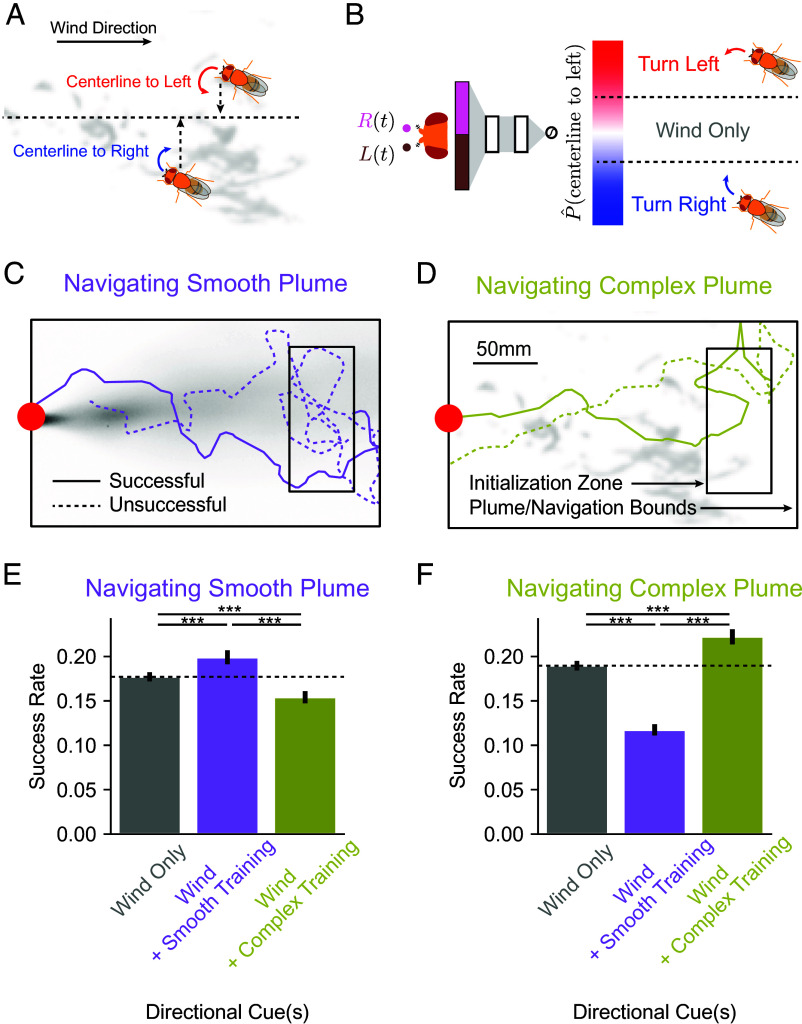
Sensitivity to plume-appropriate directional odor features improves wind-based olfactory navigation strategies. (*A*) Simulated agents navigated smooth or complex plumes. Navigation choices were made using the wind direction and estimates of the direction to the plume centerline. (*B*) Bilateral signals detected by the agent were passed through a network model (*left*, see Fig. 3) to obtain an estimate of the probability that the centerline was to the agent’s left or the agent’s right. If the network model only weakly predicted the centerline direction, agents turned upwind or downwind according to a previously published model that does not account for bilateral directional cues. If network prediction strength exceeded a threshold, agents turned toward the estimated direction to centerline. (*C* and *D*) Example successful and unsuccessful trajectories of agents navigating the smooth (*C*) or complex (*D*) plume, using the smooth-trained or complex-trained network model respectively. All agents were initialized in a box located in the crosswind plume center, with a 112 mm width and extending 200 to 250 mm downwind of the source (10 mm radius, red dot). Successful agents reached a 10 mm-radius goal at the plume’s source. Unsuccessful agents either reached a maximum of 90 s search time without reaching the source (*C*) or traveled upwind beyond the goal without entering it (*D*). (*E* and *F*) For agents navigating the smooth plume (*E*), or complex plume (*F*), success rates for baseline navigators lacking bilateral features (*gray*), with a bilateral, centerline-predicting network trained in the smooth plume (*purple*), or with a bilateral, centerline-predicting network trained in the complex plume (*gold*). Error bars are 95% CI. Pairwise comparisons of success rate assessed by z-tests (n = 10,000 trajectories per condition; *** indicates *P* < 0.001).

In the simulation, agents navigate odor plumes by alternating straight forward movement with instantaneous turns. Turns occurred stochastically at a constant rate (4/3 Hz). When turns occurred, their magnitude was normally distributed with a mean of 30° and SD of 8°, as described previously (28). Agents acting on the wind-only model only know about odor timing and wind direction (*SI Appendix*, Fig. S4*A*), but agents acting on the augmented model were given the ability to use the trained DNM networks to extract directional cues from their bilateral odor histories in order to choose between clockwise and counterclockwise turning (Fig. 4 *A* and *B*). If the network provided only weak evidence about the centerline direction, these agents used the wind-only strategy. If the network provided strong information about the centerline direction, the agents turned left or right according to the estimated direction to centerline (Fig. 4*B*). This decision-making strategy was adapted from a prior study (27). All agents were initialized randomly inside a box near the back of the plume. They were considered successful if they entered a 10 mm radius circle at the odor source (Fig. 4 *C* and *D*). If they failed to enter this region in 90 s, or if they walked upwind past the source, they were considered unsuccessful.

Performance of the wind-only model was similar across plumes (gray bars in Fig. 4 *E* and *F*). In the smooth-plume, agents using the augmented strategy with the smooth plume-trained dense network significantly outperformed agents using the wind-only strategy (Fig. 4*E*). Similarly, in the complex plume, agents using the augmented strategy with the complex plume-trained dense network also significantly outperformed agents using the wind-only strategy (Fig. 4*F*). However, agents using the augmented strategy with plume-inappropriate computations—either smooth plume-trained networks during complex plume navigation or complex plume-trained networks during smooth plume navigation—performed worse than agents using the wind-only strategy. Across both plume types, bilateral computations predicting the centerline enabled more successful navigation than strategies based only on odor timing and the wind direction; however, using a plume-appropriate computation was critical to this improvement.

### Different Directional Odor Cues Predict Fly Crosswind Turning in Different Plume Types.

**Fig. 5.**
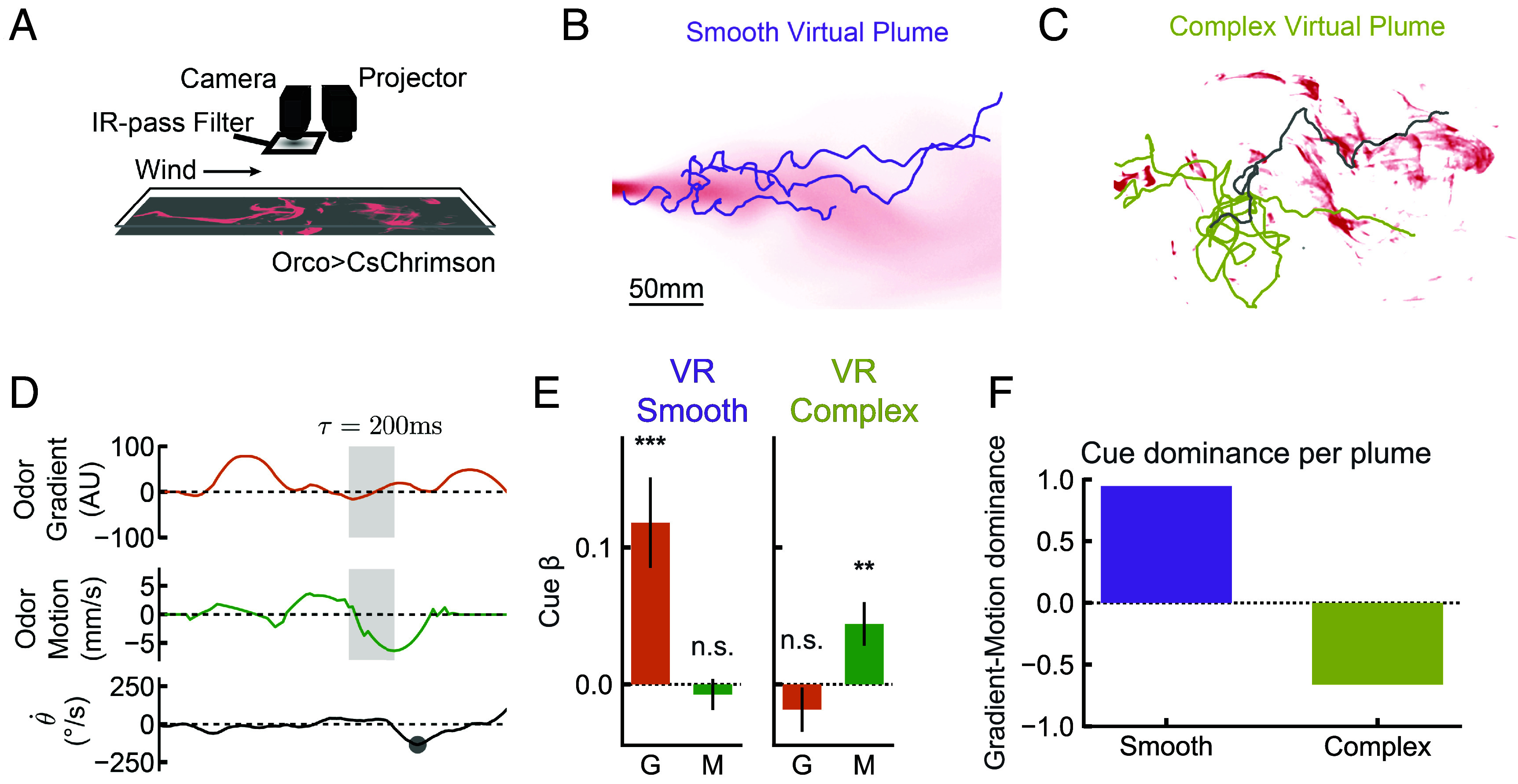
Flies make turns consistent with plume-appropriate directional odor cues. (*A*) Apparatus uses a projector to generate spatiotemporally patterned olfactory stimuli in an arena of walking flies. Olfactory stimuli were combined with 100 mm/s laminar airflow across conditions. (*B* and *C*) Example trajectories from flies navigating the smooth (*B*) or complex (*C*) virtual plumes. (*D*) A time series from one highlighted trajectory [black path segment in panel (*C*)], plotting the fly’s angular velocity (black), odor gradient (orange), and odor motion (green) over several seconds. Saccade-like turns were detected as peaks in turn speed (gray dot). Gray boxes represent 200 ms stimulus regions used to predict the direction of the upcoming turn. (*E*) Bar plots of logistic‐regression coefficients (± cluster-robust SE) for flies navigating the smooth plume (*Left*) or the complex plume (*Right*). (*F*) Cue-specific dominance of model explanatory power. A score of 1 indicates that model predictions are informed only by the gradient cue; a score of −1 indicates that model predictions are informed only by the motion cue. ** indicates *P* < 0.01; *** indicates *P* < 0.005.

Bilateral gradient and motion cues drive orienting responses in fruit flies (27, 49–51, 61) and other animals (7, 9, 13, 14, 54). After observing that these two cues have different utility in our smooth and complex plumes, we wondered whether animal behavioral responses to these cues depend on their navigation environment. Specifically, we sought to quantify the relationship between these cues and crosswind turning in flies navigating smooth and complex odor plumes under otherwise identical conditions. To present the plumes precisely, we used structured optogenetic olfactory stimulation instead of real odors, which are difficult to control. We optically projected our model plumes onto a navigation arena while flowing laminar air parallel to the plume centerline (Fig. 5*A*) (27, 61). We introduced flies into the arena that expressed CsChrimson in their olfactory neurons (*SI Appendix*, *Supplementary Methods*), thereby making the olfactory neurons light sensitive (27, 40). Importantly, our use of laminar air across plumes isolates the role of olfactory computation in navigation. However, it also eliminates natural relationships between local airflow fluctuations and odor dynamics. It remains unknown whether and how flies might use these relationships; our stimulus may destroy multimodal cues that help flies find odor sources in the wild.

We tracked the trajectory and orientation of flies as they navigated the smooth and complex plume stimuli (Fig. 5 *B* and *C* and *SI Appendix*, *Supplementary Methods*). To estimate the gradient and motion signals that the flies experienced as they navigated the arena, we combined an estimate of each fly’s antennal location with a record of the projected stimulus (Fig. 5*D*). From each fly’s rotational velocities, we identified saccade-like turning events, which reoriented flies by ~30° in the clockwise or counterclockwise direction, consistent with prior work (Fig. 5*D* and *SI Appendix*, Fig. S5 *A*–*C* and *Supplementary Methods*) (28).

To isolate the relationships between olfactory directional cues and turns directed by the wind, we selected time windows when flies were facing upwind (±20 degrees; see ref. 27). At these moments, rotations serve primarily to orient these flies in one crosswind direction or the other. We further restricted our analysis to flies that were engaged in upwind tracking in the bulk of the plume (*SI Appendix*, *Supplementary Methods*). For turns that satisfied these criteria, we used stimulus features 50 ms prior to turn onset to predict turn direction via logistic regression (permitting us to detect the fast reaction times reported in refs. 51 and 62). In the smooth plume, the gradient cue but not the motion cue was a significant predictor (Fig. 5*E*); conversely, in the complex plume the motion cue was a significant predictor, but the gradient cue was not (Fig. 5*F*).

This pattern of weights provides evidence that flies use reliable bilateral cues across different plume environments. The relatively small weight sizes reflect the overall difficulty of predicting fly turn direction, a challenge that may partly reflect limitations of our optogenetics setup (see Discussion). To normalize for overall predictive performance, we sought to further characterize the predictive contribution of each bilateral cue individually relative to the total predictive value of both cues. We constructed a cue dominance score which is 1 when the gradient cue completely accounts for the ability of bilateral cues to predict turning, and −1 when the motion cue completely accounts for this ability (*SI Appendix*, Fig. S5 *D* and *E* and *Supplementary Methods*). This measure indicates that the gradient cue dominated in the smooth plume, while the motion cue dominated in the complex plume (Fig. 5*F*).

Last, we asked whether our results imply that flies alter their turning direction algorithm across environments, or whether a single turning direction algorithm could also generate our observations. Temporal statistics vary across these plumes (27, 28, 36, 40, 43) (*SI Appendix*, Fig. S1) so we investigated whether averaging cues over time could mediate the patterns we observed. To do this, we simulated agents navigating in both plumes using a single turn direction algorithm that relied on a fixed linear combination of a time-averaged gradient cue and a time-averaged motion cue (*SI Appendix*, *Supplementary Methods*). Mirroring our analysis of the behaving flies, we used a logistic regression to predict the turn direction of upwind facing agents. We found that gradient cues were weighted more strongly in the smooth than in the complex plume; conversely, motion cues were weighted more strongly in the complex than in the smooth plume (*SI Appendix*, Fig. S5 *F*–*H*). This suggests that time-integration of directional odor information could account for our behavioral results without requiring an explicit, externally triggered switch in the underlying algorithm.

## Discussion

Odor gradient and odor motion sensing have been observed in multiple species, including both invertebrates (6, 7, 27, 49–52) and vertebrates (9, 13, 14). We have shown that these cues can provide complementary information about the plume centerline direction, with differential reliability in the two plumes we considered here. Optimizing neural network models to determine centerline direction led these models to sense gradient and motion features in the smooth and complex plumes, respectively. When embedded in navigating agents, these feature-sensing models enhanced navigation performance only when navigators use them in the appropriate, plume-specific context. Fruit flies navigated virtual odor plumes in a manner that was consistent with these predictions: In the smooth plume, gradient cues better predicted flies’ orienting decisions; in the complex plume, motion cues better predicted flies’ orienting decisions.

Bilateral sensing can support navigation in different environments, in part because gradient and motion offer independent, complementary information to navigators. Recognizing this functional distinction may advance our understanding of bilateral odor processing principles in the brain. In the fruit fly, several neural pathways combine olfactory information across hemispheres, including olfactory receptor neurons themselves, which project bilaterally to the antennal lobes (51). Recent experiments indicate that, downstream of the olfactory receptor neurons, projection neurons are sensitive to differences in odor intensity across the antennae (52). Several lateral horn neurons also project bilaterally (63), and one such cell type has been implicated in gradient-driven behavior (61). Although past research in these circuits has focused on gradient-coding (51, 52, 61), our work suggests that spatiotemporal coding principles in these and other bilaterally integrating olfactory circuits may support sensing multiple, distinct bilateral cues. An interesting dimension for future research will be to understand whether optimized bilateral olfactory computation differs systematically as sensor spacing changes. Here, we found that the spatial organization of gradient and motion cues in the complex plume remained consistent when the spatial resolution was lowered over a fivefold range or when the temporal resolution was reduced from 17 ms to about 150 ms (*SI Appendix*, Fig. S1 *F* and *G*). More generally, different sensor separation might enable detection of gradient and motion signals with different characteristic length scales and time scales. It is interesting that insects, including fruit flies, bees, cockroaches, and locusts are known to actively change the spacing of their antennae (64–67).

In this work, we tested behavioral predictions that we made in part by optimizing neural networks to solve navigational tasks using natural odor statistics. Goal-directed optimization of networks over natural stimuli is a powerful tool for understanding neural circuits (68). In *Drosophila*, it has been used to explain functional properties of visual circuits (56, 69–71). Here, this approach provided a normative rationale for sensing both odor gradient and odor motion cues to resolve the direction to centerline across different plume conditions. This approach also provided trained filters that can be interpreted as a hypothesis about neural computation. In vision, similar optimization methods have produced neural networks with functional properties that predict experimentally measured neural responses (56, 71). The filters trained in the complex plume exhibit a structure that is like the classical BL model of visual motion detection (57), combining immediate sensitivity to stimulation at one location with delayed, opposite-sign sensitivity to stimulation at a neighboring location (Fig. 3). Notably, differences in input statistics across plume types generated very different learned network architectures. Evidence in flies and other animals suggests that visual scene statistics strongly influence algorithms for visual motion detection (69, 72–81). Exploring how the olfactory system is for adapted processing odor signals in these diverse plume contexts may be an especially fruitful avenue for understanding complex neural computation.

One limitation of our study is its focus on two specific plumes generated through laboratory methods. These plumes do not capture the full range of plume variation that fruit flies are likely to experience in the wild, and we cannot predict gradient and motion use across this entire range. While we do not claim that our examples are the natural ends of a physical spectrum, they differ along navigation-relevant dimensions, like odor encounter frequency, that can align with physical parameters like wind speed (*SI Appendix*, Fig. S1 *A*–*E*).

Adding bilateral detection to our wind-only agent-based model, which was shown previously to reproduce navigational behavior across a wide range of signal frequency and duration (40, 43), improved performance by ~10% of baseline levels, in line with previous results about the contribution of gradient sensing to a model based on smooth plume navigation only (36). This modest gain in performance is not surprising. During plume navigation the dominant behavior is to move upwind while maintaining contact with the odor. Crosswind navigation is secondary and helps improve performance by reducing search time in the crosswind direction.

In our experiments the total bilateral cue predictivity was dominated by the gradient cue in the smooth plume and by motion cue in the complex plume. However, bilateral cues only slightly modulated turn direction overall. Multiple factors likely affect the magnitude of the predictivity of gradient and motion signals. Unlike in experiments that present simplified gradient (51) and motion (27) stimuli, responses to gradient and motion cues during plume navigation are expected to be less reliable since the spatiotemporal structure of the odor signal is much more complicated and wind direction dominates (28, 36). In addition, we presented laminar airflow in both our conditions, which allowed us to isolate olfactory-driven behaviors but may have broken potential multimodal computations that rely on correlations between local airflow and odor dynamics. Hence, our results likely represent a lower bound on the influence of motion and gradient cues perpendicular to the plume centerline.

Broadly, our work indicates that directional odor cues can inform crosswind navigation across two very different plumes. This insight motivates broad avenues for theoretical and empirical research into odor navigation. Crosswind navigation appears more sophisticated and better informed by directional odor cues than previously realized. In diverse, naturalistic plumes, bilateral odor sensing provides more information than just the timing cues that gate upwind turns. Our results are consistent both with flies using a single fixed algorithm—as exemplified by our cue-smoothing agents—or with flies actively adjusting their bilateral cue use strategy. An important future direction will be to test these alternatives experimentally. Our simulation specifically motivates an investigation of olfactory cue integration timescales.

Bilateral odor sensing is widely conserved and supports navigation in invertebrates (6, 7, 27, 49–52, 82) and in vertebrates (9, 13, 14, 53, 54), including in humans (15). Thus, bilateral and directional odor cues may be integral to odor plume navigation across animals. Moreover, our MNM is among the simplest that can extract gradient and motion information from bilateral time series of odor intensities. This model and others we studied are not fly-specific, beyond the spacing of the bilateral sensors. As a result, our computational approaches and results seem likely to be relevant to other species that use bilateral sensing for olfactory navigation.

## Methods

We analyzed two experimentally derived odor plume conditions, a smooth plume and a complex plume, to compare the directional information carried by bilateral odor gradient and odor motion cues. Using simulated bilateral sampling, we quantified these cues and evaluated their ability to predict the direction to the plume centerline with logistic regression models. We also trained bilateral neural network models on left and right odor histories to infer centerline direction and then incorporated those models into agent-based navigation simulations to test whether plume-matched bilateral computations improve source-finding performance. For behavioral validation, we tracked walking *Drosophila* navigating optogenetically projected plume stimuli under laminar airflow, reconstructed stimulus values at the antennae, and related cue values shortly before turns to turn direction. Detailed descriptions of plume construction, feature calculations, regression analyses, neural network training, simulation parameters, fly selection criteria, turn detection, and statistics are provided in *SI Appendix*.

## Supplementary Material

Appendix 01 (PDF)

Movie S1. Example clip from the smooth plume dataset used in this study. Odor concentration decays gradually away from the plume centerline and produces relatively continuous odor signals. Related to Fig. 1B.

Movie S2. Example clip from the complex plume dataset used in this study. Odor is organized into intermittent filaments that disperse in the crosswind direction. Related to Fig. 1C.

## Data Availability

All data and code needed to reproduce the analyses in this paper are publicly available. Plume recordings, behavioral tracking data, and processed datasets have been deposited in the Yale Dataverse (https://doi.org/10.60600/YU/590UBV) (83). Analysis code and simulation scripts are available at https://github.com/emonetlab/GradientMotionMultiplePlumes.git (84). Previously published data were used for this work (36).
