# Supplementary material for: Fly navigational responses exploit plume-specific odor motion and gradient cues
Source: Proc Natl Acad Sci U S A. 2026 Jul 14;123(29):e2608896123. doi: 10.1073/pnas.2608896123 (PMC13389596; doi:10.1073/pnas.2608896123)
Supplement: Supplementary file 1 — Appendix 01 (PDF) [file pnas.2608896123.sapp.pdf]

Fly navigational responses exploit plume-specific odor motion and gradient cues

Samuel Brudner[1,2]*, Baohua Zhou[1,2]*, Viraaj Jayaram[2,3], Gustavo Madeira Santana,[1,2,4,5], John P. Crimaldi[6], Damon A. Clark[1,2,3,4,5#], Thierry Emonet[1,2,3,5#]
1 – Department of Molecular Cellular and Developmental Biology, Yale University, New Haven, CT 06520, USA
2 – Quantitative Biology Institute, Yale University, New Haven, CT 06520, USA
3 – Department of Physics, Yale University, New Haven, CT 06520, USA
4 – Department of Neuroscience, Yale University, New Haven, CT 06510, USA
5 – Wu Tsai Institute, Yale University, New Haven, CT 06510, USA
6 – Department of Civil, Environmental & Architectural Engineering, University of Colorado, Boulder, CO 80309, USA
* Equal contributors
# Equal contributors, corresponding authors: damon.clark@yale.edu, thierry.emonet@yale.edu

Supplementary Methods

Plume movies

There were two types of plumes used in this paper, and we indicated them as smooth and complex plumes, respectively. The complex plume was a 60 Hz movie with 3600 frames. We omitted the first 300 frames in order to make sure the odor plumes reached steady state and also omitted the last 1800 frames due to the gradual decaying of the intensity. Thus, only the rest 1500 frames were used. Each frame was a matrix of size 1088×1728, and each pixel was a 0.153 mm by 0.153 mm square. In the original video, the pixel values ranged from 0 to 120, and for consistency, we linearly rescaled them to the range from 0 to 255. The smooth plume was obtained from a published paper [1, 2], and we rescaled their original movie to match the time and space resolution of our sparse plume. Each frame was a matrix of size 1088×1696. The centerline of the smooth plume was set at 544 in vertical direction, while the centerline of the complex plume was set at 590 according to the exact location of the odor source.

For each movie, we calculated the averaged frame and used it to determine sampling regions and to avoid regions with averaged plume intensity below certain thresholds. After this, 1000 random locations were uniformly selected in the valid regions. For each location, we assumed there was an artificial fly with two antennae 3 pixels apart, and the signal into each antenna was filtered by a 2d Gaussian kernel with a zero mean and a standard deviation of 1.5 pixels. Each input to one antenna was a 30-timestep or 0.5s long vector. For each movie, we created two types of datasets. In the first dataset, the orientation of the fly was fixed, and the fly always faced upwind, which means that if the fly was in the lower half of the frame, the centerline was always to its right. In the second dataset, we allowed the orientation of the fly to undergo a Wiener process, but the data was collected in a way that in the last data point, the fly was facing upwind. Explicitly, the diffusion equation was

$$\frac{d\Theta(t)}{dt} = L(t)$$
$$\langle L(t)L(t')\rangle = \Gamma\delta(t - t')$$

where the constant Γ was set to a value that the square root of the mean squared angular change in 0.5s was π. In this way, for each input vector, only the last few or most recent data points were informative about the centerline direction, since the fly could face any directions for data points that were further in the past.

In **Fig. S1**, three other plumes were also obtained from a published paper [1], which used the same recording conditions for these as for the smooth plume. We applied the same interpolation to these three plumes as we did to the smooth plume, then we linearly rescaled intensities in each movie to the 1st and 99th percentile values. To characterize frequency and intermittency **Fig. S1**, we binarized all intensity values by identifying the maximum intensity detected at each pixel and setting a threshold at 40% of that value. Analysis was restricted to a spatial mask defined by applying Otsu's method with a morphology radius of 9 pixels to mean intensity maps. To avoid masking too heavily, we applied a bias value of 55 for all plumes but the complex plume, for which we used a bias of 48.

We constructed a relative scale that orders plumes by their motion-to-gradient antisymmetric organization (Fig. S1, column v). To calculate this cue antisymmetry index, we first generated an antisymmetric component map $A_{cue}(x, y)$ from the time-averaged map of each cue $M_{cue}(x, y)$ (Fig. S1, columns iii and iv): $A_{cue}(x, y) = M_{cue}(x, y) - M_{cue}(x, -y)$, where x is the downwind coordinate and y is the crosswind coordinate with the plume centerline at y = 0. The subtraction zeros out structure that is symmetric about the centerline and retains antisymmetric structure. We defined a scalar cue score for each cue and plume by averaging absolute antisymmetric magnitude in the Otsu mask used for temporal statistics: $s_{cue} = \langle |A_{cue}| \rangle_{(x,y)\in ROI}$. The cue antisymmetry index for each plume is $\log_2 \frac{s_{motion}}{s_{gradient}}$.

Logistic regression models

We engineered three distinct scalar features, and indicated them as odor sum, odor gradient, and odor motion, respectively. Suppose the filtered signals received by the left and right antenna were represented by $L(t)$ and $R(t)$, respectively, where t ranged from 0 to 29. The odor sum feature was obtained by the following equation:

$$s1 = \frac{1}{30}\sum_{t=0}^{29}(L(t) + R(t))$$

The odor gradient feature:

$$s2 = \frac{1}{30}\sum_{t=0}^{29}(L(t) - R(t))$$

The odor motion feature:

$$s3 = \frac{1}{30}\sum_{t=0}^{29}(L(t-\tau)R(t) - L(t)R(t-\tau)),$$

where τ varied from 1 to 15. In practice, $\tau = 1$ gave us the best testing results. Each of the scalar features was fed into a Logistic regression model:

$$P(\text{Centerline was on the right}) = \sigma(a \times s + b_1),$$

where $\sigma(x) = 1/(1 + \exp(-x))$ was the sigmoid function and b_1 was the intercept.

The dataset was generated with fly orientation fixed to face upwind. The training data contained 2,400,000 samples, and the testing data contained 600,000 samples. For each of the features, the logistic regression models were trained on 100 independently generated datasets to get enough statistics. The loss function was cross entropy.

Artificial neural network model (ANN)

We built two types of ANNs: minimum ANN and dense ANN. In the minimum ANN model, there were two filters f_1 and f_2, and each was a vector with length 30. These 60 values and one bias term were the only trainable parameters in the model. The model was built as follows:

$$P(\text{Centerline was on the right}) = \sigma[ReLU(f_1^T L + f_2^T R + b_2) - ReLU(f_1^T R + f_2^T L + b_2)],$$

where $ReLU$ was the rectified linear unit function and b_2 was the intercept. The learning rate was set at 10^{-4}, the batch size was 500 and the total number of training epochs was 500.

In the dense ANN, we used a 2-layer dense neural network with each layer 20 neurons. More complex dense models might result in overfitting. In this model, the input layer had a dimension of 60, concatenating the two input signals. Each neuron in the first hidden layer received a weighted sum of the input layer, and the weights could be seen as two temporal filters, each with a length of 30. Thus, there were 20 pairs of temporal filters in total as the first layer was mapped onto the first hidden layer. The learning rate was set at 10^{-4}, the batch size was 500 and the total number of training epochs was 500.

The training data were generated with the fly orientation undergoing a Wiener process. For each type of datasets, there were 2,400,000 training samples and 600,000 testing samples. The loss function was the cross entropy.

Testing trained models on synthetic stimuli

We designed two types of synthetic stimuli to test how the trained models respond to motion or gradient features. Each stimulus contained two signals, corresponding to the left and right antennae, respectively, and the time resolution was the same as in the training samples, which was 1/60 s. For the motion-feature stimuli, left signal had random binary inputs, 0 or 1, and the right signal was the shifted version of the left signal, and the shift size varied from -6 to 6. For the gradient-feature stimuli, both left and right signals were nonnegative constants, but their separation varied from -0.5 to 0.5 and the mean of the two signals was fixed at 0.25.

Hassenstein Reichardt correlator

The simplest form of the Hassenstein Reichardt correlator can be written as

$$\hat{v} = I(x + \Delta x, t)I(x, t - \Delta t) - I(x + \Delta x, t - \Delta t)I(x, t)$$

Expanding it to the second order gives

$$\hat{v} = \left[I + \frac{\partial I}{\partial x}\Delta x + \frac{1}{2}\frac{\partial^2 I}{\partial x^2}(\Delta x)^2\right]\left[I - \frac{\partial I}{\partial t}\Delta t + \frac{1}{2}\frac{\partial^2 I}{\partial t^2}(\Delta t)^2\right]$$
$$- \left[I + \frac{\partial I}{\partial x}\Delta x - \frac{\partial I}{\partial t}\Delta t + \frac{1}{2}\frac{\partial^2 I}{\partial x^2}(\Delta x)^2 + \frac{1}{2}\frac{\partial^2 I}{\partial t^2}(\Delta t)^2 - \frac{\partial^2 I}{\partial t \partial x}\Delta x \Delta t\right] I$$
$$= -\frac{\partial I}{\partial x}\frac{\partial I}{\partial t}\Delta x \Delta t + I\frac{\partial^2 I}{\partial t \partial x}\Delta x \Delta t = -2\frac{\partial I}{\partial x}\frac{\partial I}{\partial t}\Delta x \Delta t + \frac{\partial}{\partial t}\left(I\frac{\partial I}{\partial x}\right)\Delta x \Delta t$$

the first term of which (without the factor of 2) was used to calculate the motion signals in the Figure 1. The last term in the last line is a total derivative of time, or a surface term, and usually can be omitted when the velocity is estimated as an average over the time it takes for the filament to pass over the two antennae[3].

Agent simulations

Agents navigated the plume movies described previously. They were initialized at random positions within a box downwind of the plume odor source. The box extended 200 to 250mm downwind of the source, and +/- 83mm in the crosswind direction from the plume centerline. Initial agent orientation was also randomly initialized. At each movie timestep, agents moved forward at a speed of 10mm/s in the direction of their current orientation. Turns occurred stochastically, at times generated through a Poisson process with an average rate of 4/3 Hz. We imposed reflective spatial boundaries that kept agents inside the region of the plume movie (a 'right' wall at $x = 270$; a 'top' wall at $y = 160$; a 'bottom' wall at $y = 0$). The upwind 'left' wall was not reflecting. Reaching $x = 0$ terminated trajectories. Agents navigated for 90s, or until their search was terminated by entering the goal region or passing upwind of the goal. For smooth plumes, the goal region was a 10mm circle centered at (0, 85). Because of a minor movie asymmetry, the goal region of the complex plume was centered at (0, 90).

When turns occurred, agent orientation changed by an absolute magnitude that was generated stochastically by sampling a normal distribution with a mean of 30° and standard deviation of 8°, following previous models of fly turning, and consistent with our measurements of behavior here (**Fig. S5**) [4]. At each timestep, agents sensed stimulus intensity at two antennae spaced 306µm apart along an axis perpendicular to agent orientation. In the *baseline strategy*, agents averaged these two intensity samples. This average intensity was combined with current orientation information into a goal vector according to a previously published model that describes flies' upwind orientation decisions across a diverse array of timing statistics[5]. Specifically, this average was binarized based on whether it exceeded a noise floor (5AU). When the binarized signal was above threshold, an integrator signal, R, was set to 1. When the binarized signal fell below threshold, R decayed towards 0 with a 0.97s timescale. R was combined with agent orientation to generate an upwind turning bias:
$$P_{upwind} = \frac{1}{1+e^{-(-0.49+1.5R\sin^2\theta)}}$$
(parameters from [5]). Upwind turns were generated with probability P_{upwind} (and downwind turns occurred with the complementary probability $1 - P_{upwind}$).

In the *bilateral strategy*, sampled odor intensity values at the left and right antennae were scaled and preprocessed according to our neural network procedures (see prior section). 500ms histories of each signal were passed to our trained Dense Network Models to generate an estimated

probability that the centerline was to the navigator's left side $\hat{P}(left)$. When $\hat{P}$ was near 0.5, agents using the bilateral strategy defaulted to the baseline strategy; otherwise, they turned left when $\hat{P}$ was large and turned right when $\hat{P}$ was small. Specifically, agents in Fig 4 used the baseline strategy when $\hat{P} \in (0.4, 0.6)$. They turned right when $\hat{P} < 0.4$ and turned left when $\hat{P} > 0.6$. Performance at other thresholds is given in **Fig. S4**. Baseline agents always used the baseline strategy.

Temporal smoothing agents (**Fig. S5 F-H**) computed both gradient and motion as described above. The raw cue values were both smoothed in time with a causal exponential filter (0.85s half-life) before they were linearly combined (smoothed gradient weight = 2.65; smoothed motion weight = 0.07). This sum was passed through a logistic function and the result used as $\hat{P}$ in the navigation algorithm above.

<u>Fly strains and rearing</u>

Fly rearing techniques were adapted from techniques in previous reports[5, 6]. We reared flies at 25ºC and 50% humidity under a 12 h–12 h light–dark cycle in plastic vials containing 10 ml standard glucose-cornmeal medium (Archon Scientific).

All flies in the study expressed a GMR-hid transgene that renders them blind. Optogenetic activation was achieved by expressing Chrimson (20X-UAS-CsChrimson) in Orco-expressing ORNs (Orco-GAL4). The genotypes used were: (1) w;gmr-hid;+ (gift from M. Murthy); (2) w;+;20XUAS-Chrimson (Bloomington, 55136); (3) w;+;Orco-Gal4 (gift from J. Carlson); (4) w;+.

<u>Behavioral apparatus</u>

Flies navigated an optogenetic arena identical to the prior reports[5, 6], measuring 270mm (length) by 170mm (width) by 10mm (height). Laminar air (100mm/s) was introduced through an array of straws at the upwind side of the arena. A plastic mesh at the downwind side of the arena prevented flies from escaping.

Flies in the arena were illuminated with side-mounted 850 nm IR LED strips (Waveform Lighting) and recorded through an IR filter at 60 fps with a camera (FLIR Grasshopper USB 3.0). Dry air (Airgas) was passed through an array of aligned coffee straws to create 100mm/s laminar airflow through the arena. We used a projector (DLP LightCrafter 4500) to deliver red (627nm), csChrimson-activating light stimuli throughout the walking area. Stimuli updated at 60Hz.

The stimulus presentation and data recording operations were controlled in tandem by custom software written in Python 3.6.5. More detail about the control software is available in a prior publication [6].

<u>Stimulus design</u>

We projected to-scale movies of the recorded smooth and complex plumes across the arena. Between the two plumes, data values do not reflect identical quantities (see Plume Data) and are not recorded on the same scales. We linearly scaled intensities in the smooth plume to span the range of intensities we could deliver through our projector. We used a transformation of the complex plume into projector intensities using the methods in [6]. This transformation includes a background subtraction to reduce noise in the movie, floor and ceiling thresholding of the signal, and then linear scaling to fill the projector dynamic range. The nonlinear thresholding process highlights contrast between odor filaments and interposed blank air.

Each trial lasted 2 minutes. On smooth-plume trials, we presented the first 2 minutes of the smooth movie; on complex-plume trials, the shorter complex movie was looped repeatedly to fill the 2-minute interval. A 1-minute rest period followed each trial. To avoid directional biases, each plume's projection was flipped across its centerline between successive trials. Individual fly cohorts experienced either smooth-plume trials or complex-plume trials, but not both.

Experimental Protocol

Between 10 and 30 females (<3 days post-eclosion) were placed in empty vials containing water-soaked cotton plugs at the bottom and top. These flies were starved for 3 days before recordings began. 24 hours before our experiments, the flies were fed 1 mM all trans-Retinal (ATR) (MilliporeSigma) dissolved in water and subsequently housed in the dark. All flies in a single starve vial were run in an experiment simultaneously.

We used the incubator light/dark cycle to identify "dawn" epochs (the first 3 hours of the light cycle) and "dusk" epochs (the last 3 hours of the light cycle). We ran all experiments inside these circadian windows, when fly activity peaks.

At the start of each experiment a fly cohort was aspirated into the back of the arena through a sealable hole in the arena's ceiling. Flies acclimatized to the environment, including the laminar airflow, for 2 minutes prior to experiment onset. Each fly cohort navigated either smooth plume trials or complex plume trials. An experiment on one fly cohort consisted of 8 navigation trials.

Preprocessing

We identified fly head and abdomen positions from recorded videos using SLEAP [7]. We smoothed position trajectories using a 4th order Savitzky-Golay filter with a 21-sample window (0.3s). The analytic derivatives of these polynomial fits were used to quantify x and y velocities. To determine fly orientation, we first estimated its body outline using scikit-image's "canny" function. We found the best-fit ellipse approximation to this outline and treated the major axis as the head-to-tail axis. When the fly was moving, our estimates of the ground velocity were used to disambiguate the head from the tail, since flies typically move forward rather than backward. When the flies were still, we used the major axis orientation that best matched the abdomen-to-head angle found through SLEAP. We used the same smoothing operations on orientation traces to obtain smoothed fly orientation and rotational velocity traces.

To estimate the stimuli flies experienced, we adapted previously published gradient and motion calculation methods [6]. We estimated the antennal sensory axis as perpendicular to the major fly orientation axis and located it 1.5mm anterior to the fly abdomen location. Following a prior procedure [6], we accessed 15 pixels along the antenna axis of the fly at each time point and used the slope of projector intensity values across this slice as our gradient measure. Odor motion was estimated by cross-correlating slices from consecutive frames, identifying the lateral offset that maximized the correlation, and converting that offset to a velocity-like measure based on calibration (mm per pixel) and sampling frequency. These signals were then smoothed using an exponential filter ($\tau = 200\text{ms}$) and z-scored for subsequent analysis.

Behavioral Analysis

We located candidate turning events by finding local maxima in absolute angular velocity. A density-based clustering algorithm (DBSCAN algorithm; eps=0.1, min_samples=900) was applied to the joint distribution of log-transformed peak amplitude and peak height. We treated a dense region in the resulting distribution as exemplary saccade-like turns after inspecting the surrounding trajectories and the associated heading changes (**Fig. S5**).

For analysis, we retained only turns that met the following conditions:

1. Upwind orientation: Heading angles within ±20° of directly upwind (160–200°).
2. Active upwind walking: Positive horizontal velocity as determined via a fourth-order Butterworth filter (cutoff=0.2 Hz) applied to vx.
3. Arena region: Horizontal positions 3cm<x<22cm, with vertical position y contained within a 3cm band around the plume center line.

We modeled turn direction (left vs. right) using logistic regression. We used the smoothed and z-scored estimates of olfactory cues intensity, gradient, and motion as predictors. Specifically, we used their values 50ms prior to turn onset (time of crossing 20% turn speed peak height on the rising edge of the peak). The timescale of 50ms was chosen to reflect an approximate minimal response time to bilateral odor cues [8]. Intensity was included to account for an observed bias exhibited by upwind-facing animals for rightward turns, towards the projector mount location, with increased overall projected light intensity. Separate models were fit for each plume type. Observations from each fly trajectory were grouped to compute cluster-robust (trajectory-level) standard errors in the Statsmodels logistic framework. This approach provided estimates of how variation in odor-derived cues influence the log odds of turning left vs right.

In order to quantify the predictive contribution of gradient and motion relative to the overall predictive contribution of bilateral cue information, we compared predictions across a model family derived from the models described above. Specifically, we compared the log likelihood of fly turns under each of 4 models (See **Fig. S5 D,E**): a full model that contained all cues as described above; a base model that contained no bilateral cue predictors; a gradient model that contained no motion predictor; and a motion model that contained no gradient predictor. We quantified the *total* predictive contribution of bilateral information as the log likelihood difference between the full and base models. We quantified the *differential* predictive value of the gradient and motion cue as the difference in log likelihood between the gradient and motion models. The ratio of the differential contribution and the total contribution can vary between 1

and -1. A value of 1 indicates that the total predictive contribution of bilateral cue information exclusively reflects the predictive contribution of the gradient cue; a value of -1 indicates that the total exclusively reflects the predictive contribution of the motion cue.

Supplementary Figures

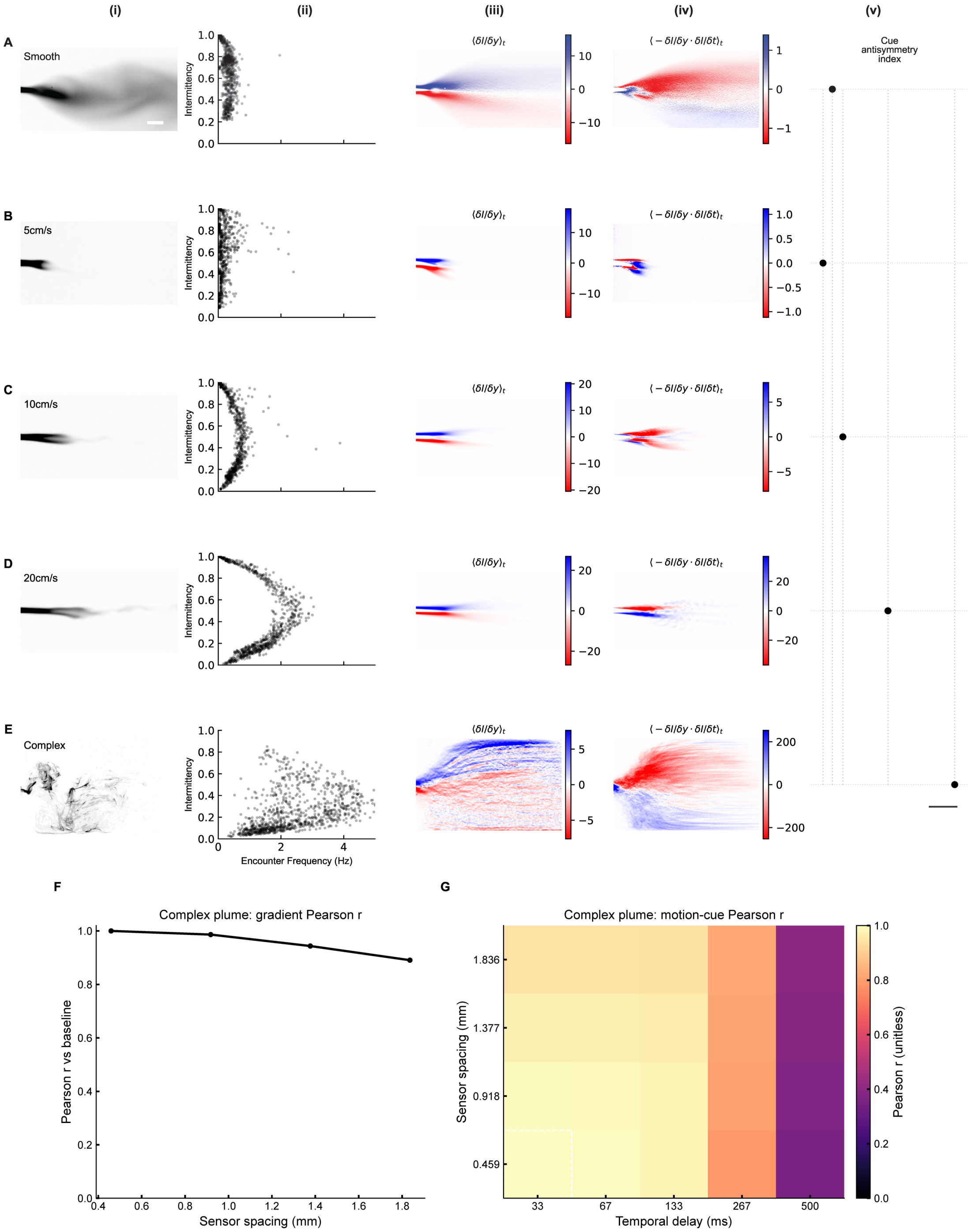

Figure S1: Odor timing and directional information across multiple plumes and sampling strategies. (A-E) Comparison of our smooth plume (A), free-stream odor plumes at 3 downwind air flow speeds (B-D), and our complex plume (E). (i) An odor intensity snapshot for each plume. 10mm scale bar. (ii) We

binarized plume intensities using spatially local thresholds. We then calculated the frequency of onsets in the binary series at each pixel and the overall fraction of time odor was above threshold at each pixel (frequency, x axis, and intermittency, y axis, respectively). The distribution of frequency and intermittency values at slow freestream speeds was similar to the distribution of values in the smooth plume. In the fast freestream plume, intermittency was relatively lower and frequency relatively higher. In the complex plume we observed the highest arrival frequencies, which remained within bounds observed in field measurements (up to 10Hz [26]). (iii) Time average of the local gradient value in the crosswind direction. (iv) Time average of the local motion value in the crosswind direction. Color scales differ across plumes in columns (iii) and (iv) to keep weak signals visible (e.g. motion in A & B versus D & E); for a scale-independent comparison across plumes, see column (v). (v) Cue antisymmetry index for each plume (see Methods), placing each plume on a relative scale of motion-to-gradient antisymmetric organization; larger values indicate plumes with relatively larger motion vs gradient antisymmetry. Across the freestream plumes the index increases monotonically with wind speed. The smooth plume index falls near the low end of this range and the complex plume index is the highest measured. $\log_2$ unit scale bar. (F) Pearson correlation between spatial maps of mean cross-wind gradient cue in the complex plume as sensor spacing increases from 3 to 12 pixels (0.46-1.84mm). (G) Pearson correlation between spatial maps of mean crosswind odor motion cue in the complex plume as a function of sensor spacing (same as gradient) and delay (1, 2, 4, 8, 15 frames; 33 to 500ms).

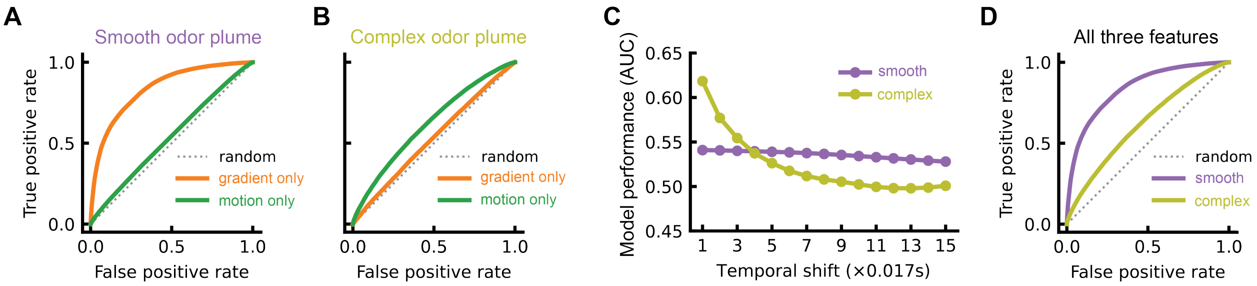

Figure S2: (A) Receiver operating curves associated with **Fig. 2B**. (B) Receiver operating curves associated with **Fig. 2C**. (C) ROC AUC scores as the functions of the temporal shift size in the engineered motion feature in both the smooth and complex plumes. (D) Receiver operating curves associated with **Fig. 2D, E**, with ROC AUC scores 0.847 (smooth plume) and 0.612 (complex plume).

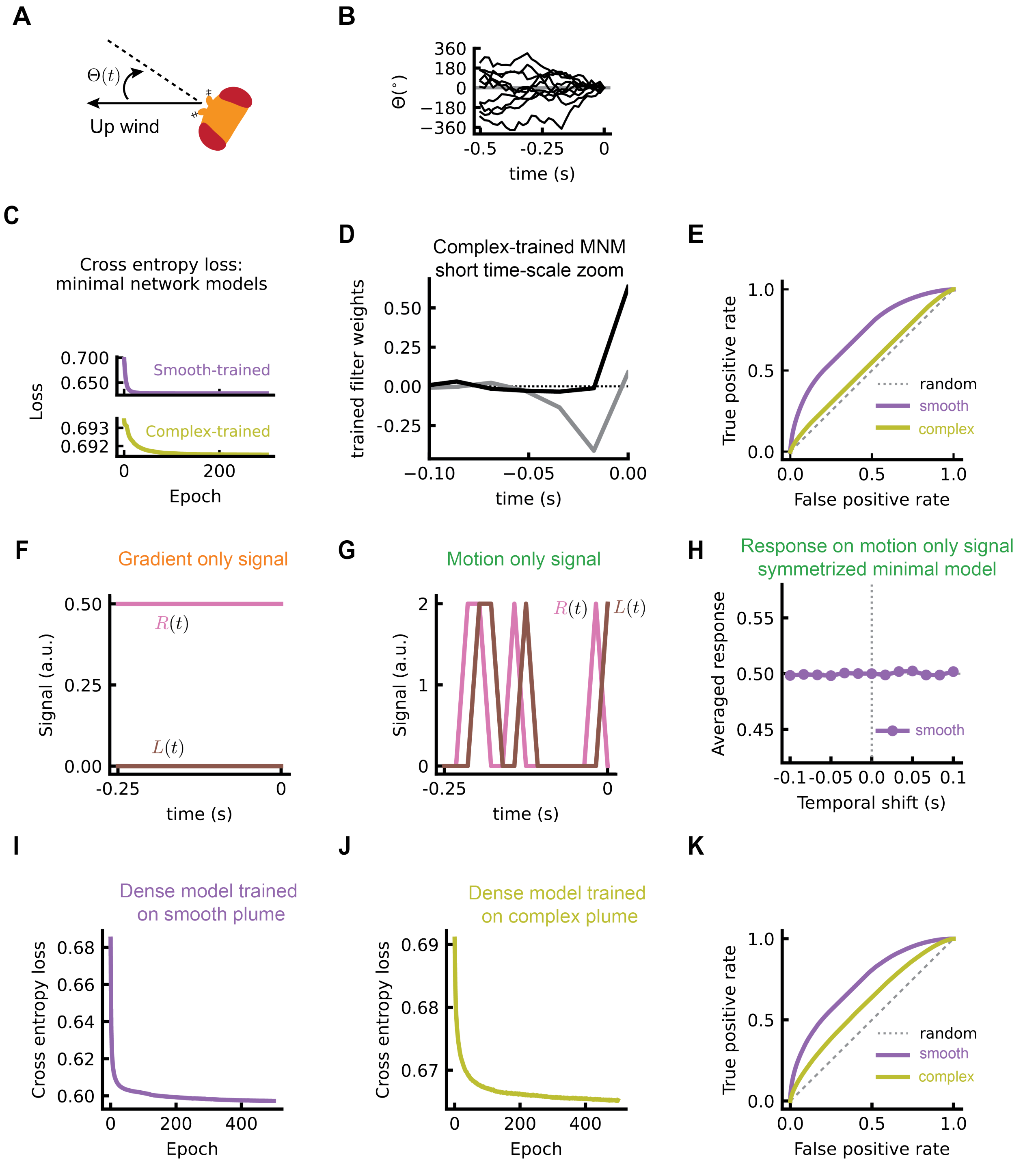

Figure S3: (A) The angle between the heading of the fly and the upwind direction is $\Theta(t)$. (B) Example trajectories of the angle $\Theta(t)$ in our simulations to generate synthetic traces for model training. At the time 0, when turning decision is made, the heading of the fly is always exactly upwind. (C) Training loss of the MNM as a function of the training epoch in the smooth (top) and complex (bottom) odor plumes. (D) First 0.1s of the minimal network model filter, trained in the complex plume. (E) Test data receiver operating curves for the MNMs trained in different plumes, with AUC scores 0.726 (smooth plume) and 0.517 (complex plume). (F) Example testing signals with only gradient information (related to **Fig. 3D, I**). (G) Example testing signals with only motion information, creating correlations between the antenna at

a single temporal offset (related to **Fig. 3E, J**, see Methods). (H) Similar to Figure 3E, but with symmetrized filters for the MNM trained in the smooth plume. (I, J) Training loss of the DNM as a function of the training epoch in the smooth (I) and complex (J) odor plumes. (K) Test data receiver operating curves for the DNMs trained in different plume types with AUC scores 0.735 (smooth plume) and 0.543 (complex plume).

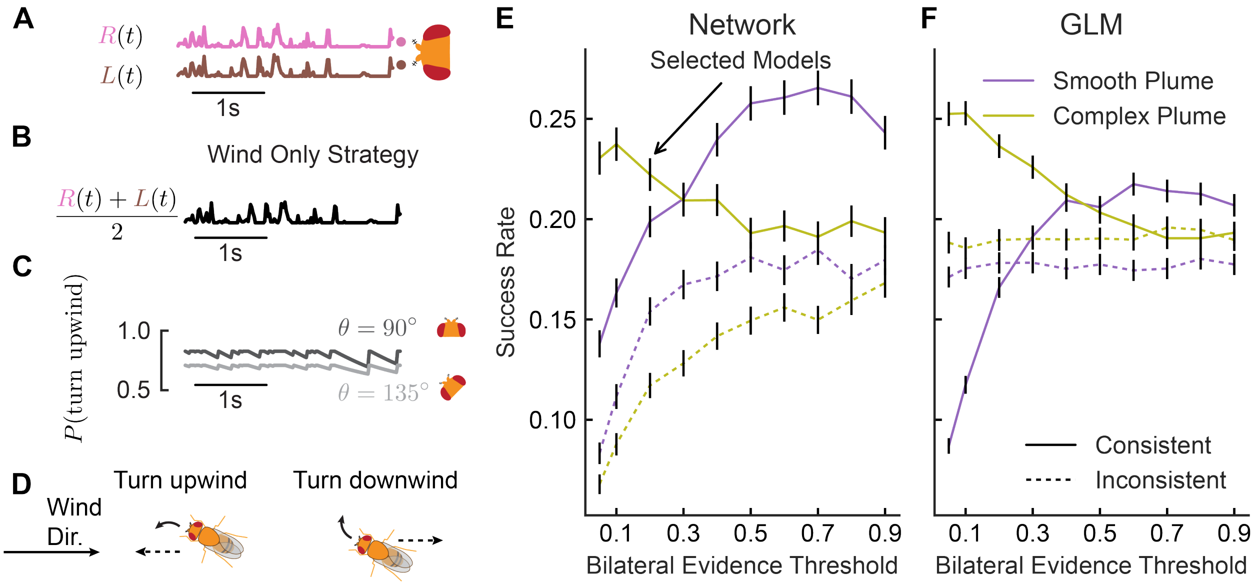

Figure S4: Outline of the baseline strategy and performances of different implementations of the bilateral strategy. (A) Example traces of bilateral signal intensity in a simulated agent. (B-D) In a baseline strategy that only uses wind as a directional cue, agents average the signal across the antennae (B). The agents calculate an upwind goal probability (C) based on this average intensity and on their current orientation. (D) After drawing an upwind or downwind goal direction on the basis of this computed probability, agents turned upwind or downwind according to the draw. This previously described model increases upwind turning in high intermittency and high frequency environments, and was parameterized on the basis of prior work [40, 43]. (E-F) Agents that calculated directional odor features used the baseline model when the output of their directional odor model was weak. We tested performance across a range of thresholds on the strength of the bilateral evidence, both in agents that used neural network calculations (E) and in agents that used a GLM using predefined directional features (gradient and motion as in **Fig. 2**) (F) as predictors of the centerline direction. Although these thresholds affect absolute performance levels, across thresholds agents using consistent features (the output of smooth-trained networks or smooth-trained, feature-based GLMs in the smooth plume; the output of complex-trained networks or complex-trained, feature-based GLMs in the complex plume) outperformed agents using inconsistent features (the output of complex-trained networks or complex trained GLMs in the smooth plume; the output of smooth-trained networks or smooth-trained GLMs in the complex plume).

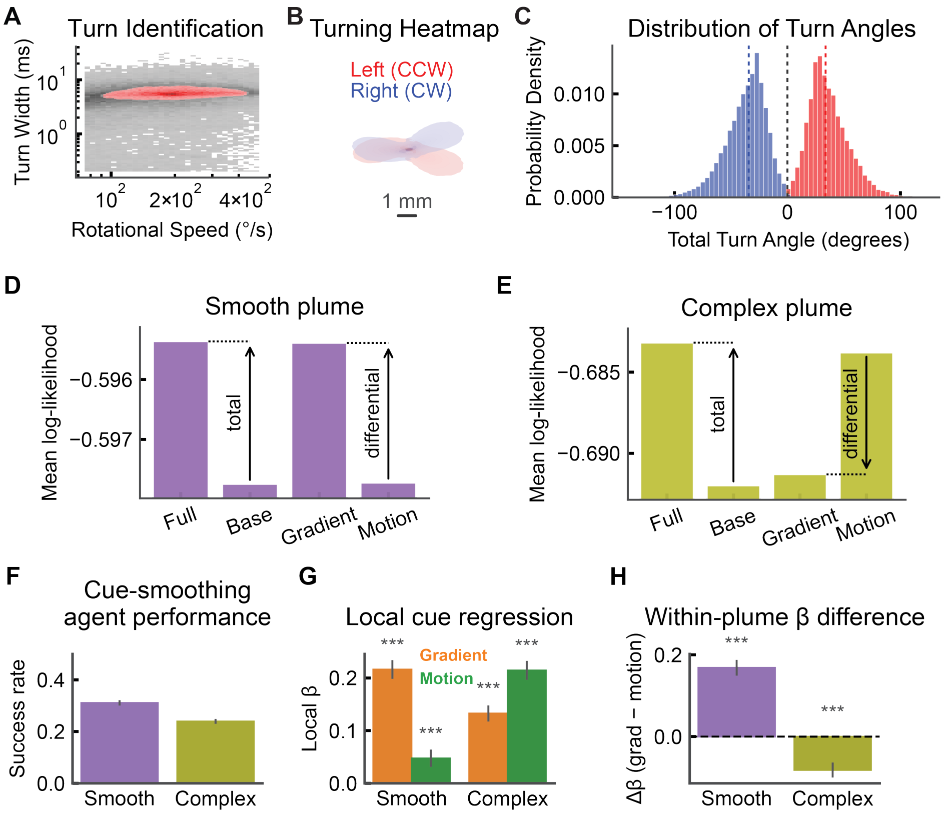

Figure S5: Analysis of cue-response relationships. (A-C) Detection and validation of saccade-like turns. (A) Histogram of the width (at half height) and amplitude of peaks in flies' instantaneous rotational speed (log scale). An over-represented, dense region was isolated using a density based cluster-finding algorithm (DBSCAN, maroon) [82]. (B) Density of fly position in the vicinity (+/- 200ms) of peaks labeled as turns. Positions are translated and rotated so they have identical spatial coordinates and orientations at the moment of the detected peak in rotational speed. Trajectories are colored according to the sign of the rotational velocity at the peak rotational speed. This density map represents the mean trajectories associated with left and right saccades. (C) Angular displacement during the identified turn events, colored according to the sign of the rotational velocity at the peak rotational speed. Median values (dashed lines; -34.2°, +33.8°) are similar to turn displacements reported in flies navigating plumes of smoke[28]. (D-E) Log likelihood of turn decision under each member of hierarchical model family for flies navigating the smooth (D) or complex (E) plume. The full model includes both gradient and motion terms; the null model contains neither; the gradient (motion) model does not contain the motion (gradient) predictor. The difference in log likelihood between the base and full model is the *total* bilateral cue predictive value. The difference in log likelihood between the gradient and motion models is the *differential* bilateral cue predictive value. We calculate cue dominance as the differential predictive value relative to the total predictive value (see Figure 5). A value of 1 means the entire predictive value of bilateral sensing can be attributed to gradient sensing; a value of -1 means the entire predictive value of bilateral sensing can be attributed to motion sensing. (F-G) Analysis of cue-smoothing agent navigators. (F) Per-plume success rate of a single agent that temporally averages gradient and motion cues before combining them with fixed weights. (G-H) Local gradient and motion values at decision time predict turn direction via logistic regression. The fitted cue weights (G) differ across plumes. (H) Specifically, in the smooth plume the gradient cue weight is larger than the motion cue weight and vice versa in the complex plume. (***; $p < 0.005$).

Supplementary Movies

Movie S1: Example clip from the smooth plume dataset used in this study. Odor concentration decays gradually away from the plume centerline and produces relatively continuous odor signals. Related to Fig. 1B.

Movie S2: Example clip from the complex plume dataset used in this study. Odor is organized into intermittent filaments that disperse in the crosswind direction. Related to Fig. 1C.

References

1. Connor, E.G., M.K. McHugh, and J.P. Crimaldi, *Quantification of airborne odor plumes using planar laser-induced fluorescence.* Experiments in Fluids, 2018. **59**: p. 1–11.

2. Alvarez-Salvado, E., et al., *Elementary sensory-motor transformations underlying olfactory navigation in walking fruit-flies.* Elife, 2018. **7**.

3. Sinha, S.R., W. Bialek, and R.R.R. van Steveninck, *Optimal Local Estimates of Visual Motion in a Natural Environment.* Phys Rev Lett, 2021. **126**(1): p. 018101.

4. Demir, M., et al., *Walking Drosophila navigate complex plumes using stochastic decisions biased by the timing of odor encounters.* Elife, 2020. **9**.

5. Jayaram, V., et al., *Temporal novelty detection and multiple timescale integration drive Drosophila orientation dynamics in temporally diverse olfactory environments.* PLoS Comput Biol, 2023. **19**(5): p. e1010606.

6. Kadakia, N., et al., *Odour motion sensing enhances navigation of complex plumes.* Nature, 2022. **611**(7937): p. 754–761.

7. Pereira, T.D., et al., *SLEAP: A deep learning system for multi-animal pose tracking.* Nature methods, 2022. **19**(4): p. 486–495.

8. Gaudry, Q., et al., *Asymmetric neurotransmitter release enables rapid odour lateralization in Drosophila.* Nature, 2013. **493**(7432): p. 424–8.
